# WGA is a probe for migrasomes

**DOI:** 10.1038/s41421-018-0078-2

**Published:** 2019-02-26

**Authors:** Lilian Chen, Liang Ma, Li Yu

**Affiliations:** 1 State Key Laboratory of Membrane Biology, Tsinghua University-Peking University Joint Center for Life Sciences, School of Life Sciences, Tsinghua University, 100084 Beijing, China; 2 Department of Molecular and Cellular Biology, Howard Hughes Medical Institute, University of California, Berkeley, CA 94720 USA; 3 Present Address: Department of Molecular and Cell Biology, University of California, Berkeley, CA 94720 USA

**Keywords:** Cell biology, Biological techniques

Dear Editor,

During cell migration, cells leave behind long tubular retraction fibers. Previously, we identified that vesicles with diameters up to 2 µm, which we called migrasomes, grow at the tips and intersections of retraction fibers^1^. We found that the migrasomes contain cytosol, which can be released into the extracellular environment or directly taken up by surrounding cells^1^. Therefore, we proposed that migrasomes may mediate cell–cell communications. We identified Tetraspanin4 (Tspan4) as a marker for migrasomes, and thus we can detect migrasomes in various types of cells and characterize the process of migrasome biogenesis. However, the limitations of using a fluorescently tagged marker protein for detection of an organelle are obvious. First, it is time consuming; transfection and expression of the marker protein takes at least 1 day before observation. Second, the transfections of many cells, especially many primary cells, are very difficult. For these cells, complicated transfection strategies (e.g., virus-mediated transfection) are required, which further increase the time and cost of migrasome detection. Finally, overexpression of fluorescently tagged protein always has the risk that the overexpressed marker protein may change the biogenesis of migrasomes, thus causing artifacts. Thus a rapid, easy, and non-interfering method is needed for detection of migrasomes.

By serendipity, we found that fluorescently tagged wheat-germ agglutinin (WGA), a lectin that binds specifically to sialic acid and *N*-acetyl-d-glucosamine^2^, labeled migrasomes in living cells (Fig. 1a). Moreover, florescence intensity analysis showed that the WGA signal on migrasomes was much higher than the WGA signal on retraction fibers (Fig. 1b, c), indicating that WGA prefers to bind to migrasomes. Finally, WGA effectively labeled migrasomes in all the cell lines we tested (Supplementary Fig. S1). Thus fluorescently tagged WGA can be used as a probe for migrasomes.

**Fig. 1:**
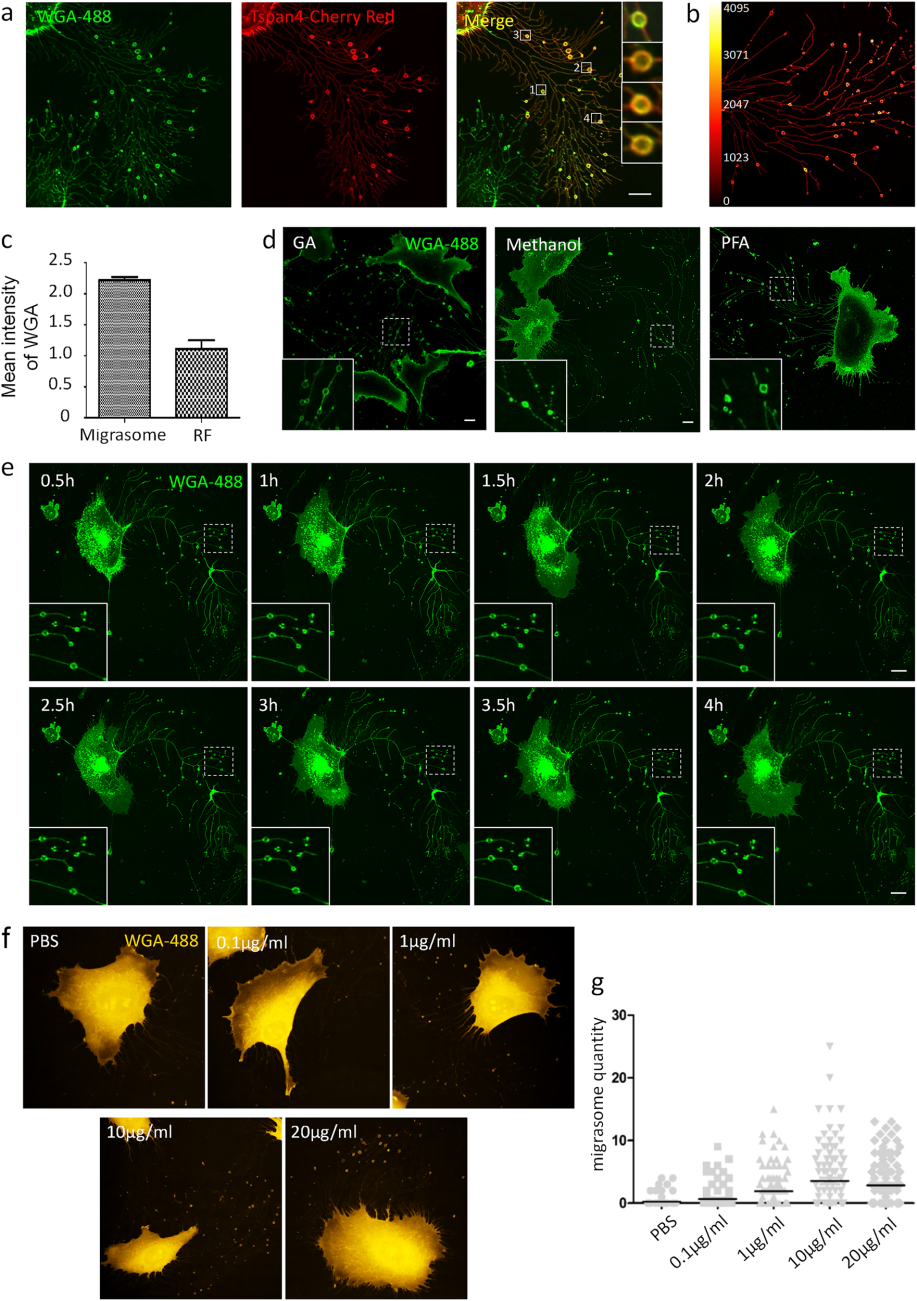
Detection of migrasomes by WGA. **a** L929 cells were transfected with Tspan-mCherry and cultured for 12 h, then stained with 1 μg/ml WGA-Alexa 488. Cells were observed by confocal microscopy. Scale bar, 10 μm. **b** L929 cells were stained with 1 μg/ml WGA-Alexa 488. Cells were observed by confocal microscopy. Enrichment of WGA-Alexa 488 on migrasomes was revealed by fluorescence intensity heat mapping. **c** Cells from **a** were measured for WGA-Alexa 488 fluorescence intensity in migrasomes and retraction fibers by the Olympus FLUOVIEW Ver.2.0b Viewer software; *n* = 450 migrasomes/retraction fibers (RFs) from three independent experiments. *t* test, *P* < 0.0001. Error bars indicate mean ± SD. **d** L929 cells were fixed with 2.5% glutaraldehyde, methanol, or 4% paraformaldehyde, then stained with WGA-Alexa 488. Cells were observed by confocal microscopy. Scale bar, 10 μm. **e** L929 cells were stained with 1 μg/ml WGA-Alexa 488, which was present in the medium during imaging. Images were captured every 30 min for 4 h. **f** L929 cells were cultured for 12 h on microwell plates coated with different concentrations of fibronectin, then fixed with 2.5% glutaraldehyde and stained with WGA. Images were taken with the Opera Phenix system. **g** The number of migrasomes per cells with different concentrations of fibronectin in **f**

Next, we investigated the optimal conditions for WGA staining of fixed cells. We fixed cells in paraformaldehyde (PFA), methanol, and glutaraldehyde (GA), then stained them with WGA. Migrasomes and retraction fibers could be preserved and labeled efficiently in GA-fixed cells (Fig. 1d); however, cells generated strong auto-fluorescence after GA fixation, which could be quenched effectively by treating the cells with sodium borohydride after fixation (Supplementary Fig. S2a). In contrast, in methanol- or PFA-fixed cells, retraction fibers and migrasomes were not preserved well (Fig. 1d). In GA-fixed cells, we found that the optimum working concentration of WGA for migrasome staining was 1 µg/ml (Supplementary Fig. S2b). Using this concentration, staining for as short as 1 min generated a strong signal (Supplementary Fig. S2c).

Next, we tested whether WGA staining can be used in time-lapse imaging. First, we stained cells with WGA, then removed the medium and washed cells with phosphate-buffered saline to reduce background fluorescence. We found that most of the WGA signal was endocytosed into the cells 1 h after labeling (Supplementary Fig. S3a). Thus this approach is not suitable for time-lapse imaging >1 h. To monitor migrasome formation for longer periods of time, we kept WGA in the culture medium during imaging. We found that this approach gave a reasonable signal-to-noise ratio and enabled us to monitor migrasomes for a much longer time (Fig. 1e). To check whether long-term exposure to WGA can affect migrasome biogenesis and cell migration, we performed time-lapse imaging and compared the migration of cells with or without WGA. We found that, although the presence of WGA slightly affected cell migration (Supplementary Fig. S3b), however, the formation of migrasomes was not affected by the presence of WGA (Supplementary Fig. S3c). Finally, we tested whether WGA staining is compatible with a high-content screening system. Previously, we showed that fibronectin increases the formation of migrasomes; however, the optimal concentration of fibronectin is not known yet. By WGA labeling, we were able to test a series of concentrations using an Opera Phenix high-content screening system, and we determined that 10 μg/ml fibronectin is the optimal working concentration for generation of migrasomes (Fig. 1f, g).

In summary, we found that WGA is a probe for convenient, rapid detection of migrasomes in both fixed and living cells.

## Supplementary information

Supplementary Information

## Supplementary information

**Supplementary Information** accompanies the paper at (10.1038/s41421-018-0078-2).
