# Supplementary material for: WGA is a probe for migrasomes
Source: Cell Discov. 2019 Feb 26;5:13. doi: 10.1038/s41421-018-0078-2 (PMC6389894; doi:10.1038/s41421-018-0078-2)
Supplement: Supplementary file 1 — Supplementary Information [file 41421_2018_78_MOESM1_ESM.pdf]

Supplementary materials

Figure S1

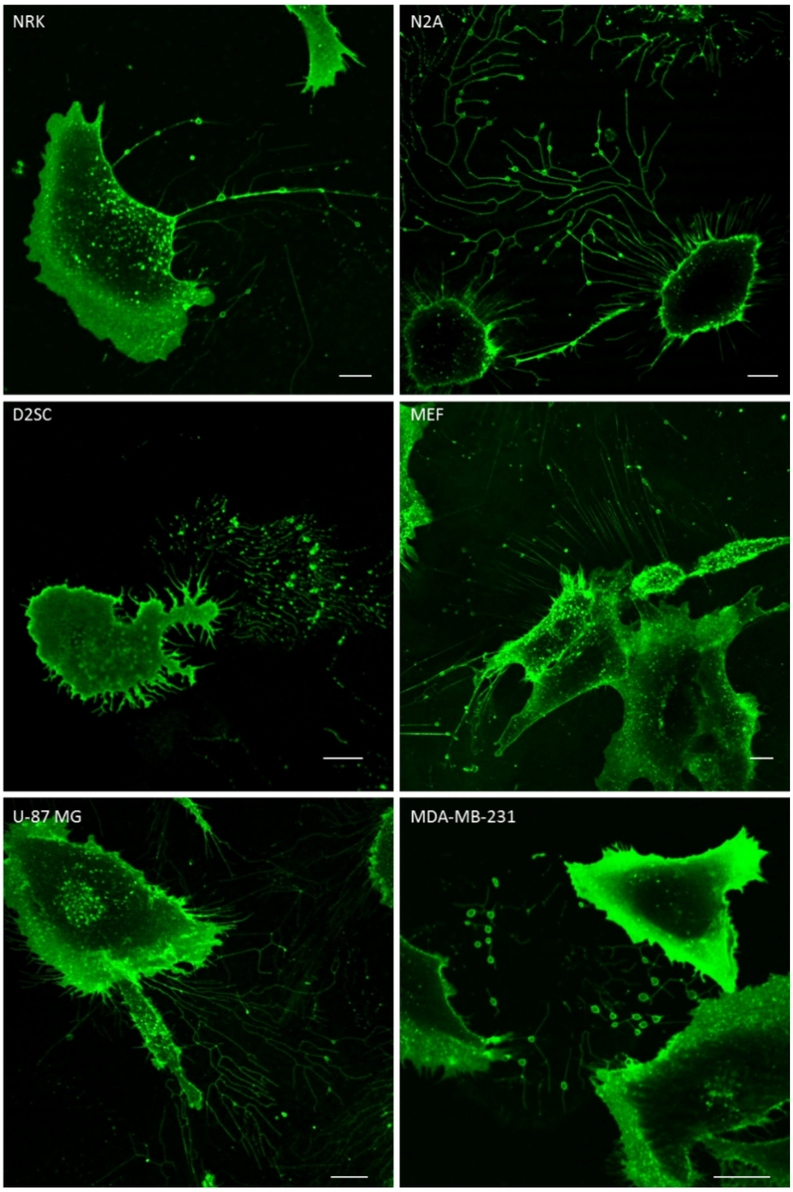

Figure S1. NRK, N2A, MEF, D2SC, U-87 MG and MDA-MB-231 cells were stained with 1 µg/ml WGA-tetramethylrhodamine. Cells were observed by confocal microscopy. Scale bar, 10 µm.

Figure S2

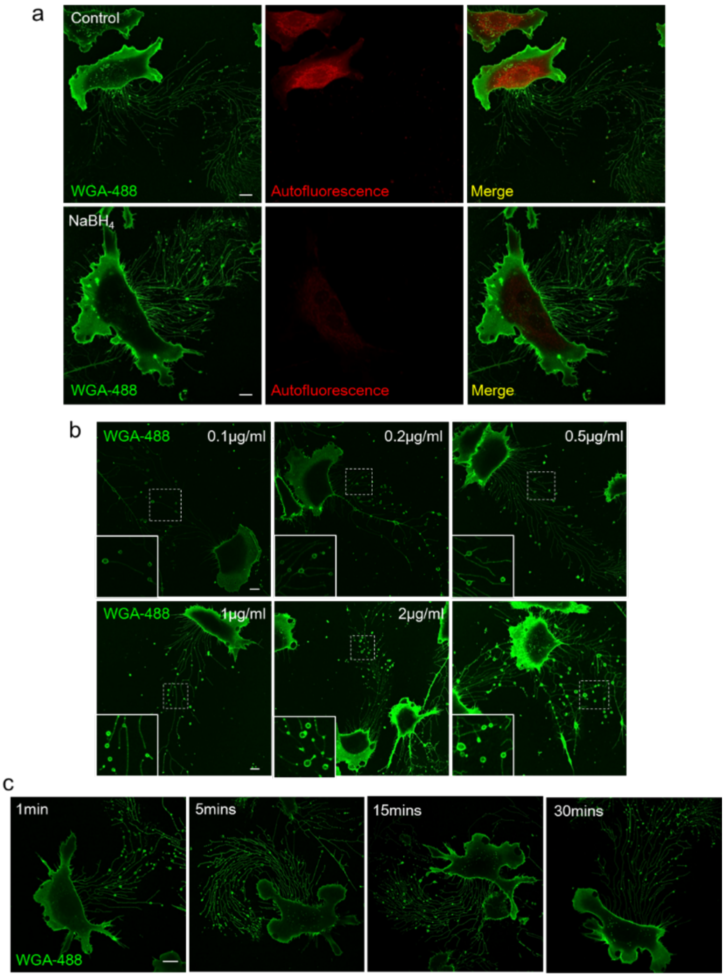

Figure S2. (a) L929 cells were fixed with 2.5% glutaraldehyde for 10 mins and stained with 1 μg/ml WGA-Alexa 488 for 10 mins, then treated with or without 1 mg/ml sodium borohydride for 7 mins. Cells were observed by confocal microscopy. Scale bar, 10 μm. (b) L929 cells were fixed with 2.5% glutaraldehyde, then stained with 0.1 μg/ml, 0.2 μg/ml, 0.5 μg/ml, 1 μg/ml, 2 μg/ml or 5 μg/ml WGA-Alexa 488 for 10 mins. Cells were observed by confocal microscopy. Scale bar, 10 μm. (c) L929 cells were fixed with 2.5% glutaraldehyde and then stained with WGA-Alexa 488 for 1 min, 5 mins, 15 mins, and 30 mins. Cells were observed by confocal microscopy. Scale bar, 10 μm.

Figure S3

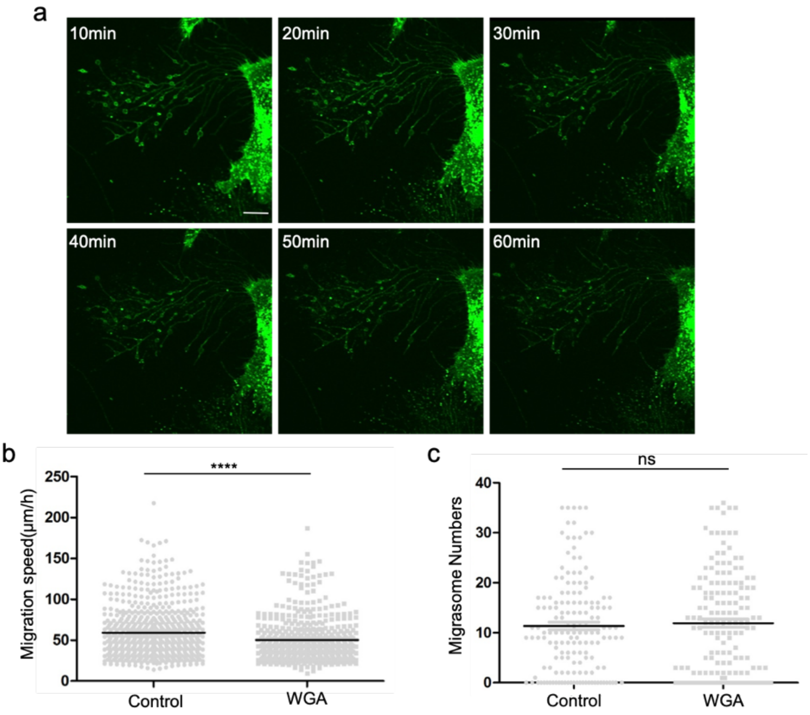

Figure S3. (a) L929 cells were stained with 1 μg/ml WGA-Alexa 488 for 5 mins, then the WGA-Alexa 488-containing medium was removed and the cells were washed with PBS to further remove WGA-Alexa 488. Images were captured every 10 mins for 60 mins. (b) L929 cells stably expressing luciferase-GFP were imaged every 10 mins for 6 hs with or without WGA-Alexa 488 (1 μg/ml), and the migration track length of the cells was measured using ImageJ. The migration speed was then calculated. n=500 cells from three independent experiments, t test P<0.0001, Error bars indicate mean±SD. (c) L929 cells were cultured for 7 hs with or without WGA-tetramethylrhodamine, then fixed with 2.5% glutaraldehyde and stained with 1 μg/ml WGA-Alexa 488. The number of migrasomes per cell was determined. n=150 cells from three independent experiments, t test P=0.6165, Error bars indicate mean±SD. Scale bar, 10 μm.

Materials and Methods

Reagents

WGA (W7024) was purchased from Invitrogen. Fibronectin (PHE0023) was purchased from Gibco. Vigofect (T001) was purchased from Vigorous. Lysosome isolation kit (LYSISO1) was purchased from Sigma-Aldrich.

Cell culture and transfection

NRK cells and L929 cells were cultured in DMEM medium (Hyclone) supplemented with 10% FBS (5% CO_2). Cells were transfected with DNA using Vigofect. Cells were seeded in a 6-well plate at a density of 6×10^5 per well and transfected with 100 μl PBS that contained 2 μl Vigofect (T001) and 2.5 μg plasmid. The transfection mix was replaced with fresh medium after 4-6 hours.

Constructs

The N-terminal mCherry-tagged Rat Tspan4 was cloned into the HindIII-AgeI sites of pmCherry-N1 plasmid using a Clone Express® II One Step Cloning Kit (Vazyme C112-02).

Live-cell imaging

The night before imaging, cells were cultured in 35 mm glass-bottom dishes coated with fibronectin. Images were acquired using Nikon A1 and Olympus FV-1000 confocal microscopes.

Statistical analysis

Two-tailed t-test was performed in GraphPad Prism. Data from 3 independent experiments were analyzed. Error bars in the figures represent the standard deviation (SD); n values are specified in the figure legends.
